# PRISM protocol: a randomised phase II trial of nivolumab in combination with alternatively scheduled ipilimumab in first-line treatment of patients with advanced or metastatic renal cell carcinoma

**DOI:** 10.1186/s12885-019-6273-1

**Published:** 2019-11-14

**Authors:** Hannah L. Buckley, Fiona J. Collinson, Gemma Ainsworth, Heather Poad, Louise Flanagan, Eszter Katona, Helen C. Howard, Geraldine Murden, Rosamonde E. Banks, Joanne Brown, Galina Velikova, Tom Waddell, Kate Fife, Paul D. Nathan, James Larkin, Thomas Powles, Sarah R. Brown, Naveen S. Vasudev

**Affiliations:** 1 Clinical Trials Research Unit, University of Leeds, Leeds, LS2 9JT UK; 2 Leeds Institute of Medical Research at St James’s, St. James’s University Hospital, Beckett Street, Leeds, LS9 7TF UK; 3 University of Leeds, Leeds, LS9 7TF UK; 4 Department of Medical Oncology, Christie Hospital, Manchester, M20 4BX UK; 5 Addenbrooke’s Hospital, Cambridge, CB2 0QQ UK; 6 Mount Vernon Cancer Centre, Middlesex, HA6 2RN UK; 7 Royal Marsden Hospital, London, SW3 6JJ UK; 8 Barts Cancer Institute, London, EC1M 6BQ UK

**Keywords:** Renal cancer, Nivolumab, Ipilimumab, Schedule, Safety, Efficacy, Randomised, Immunotherapy, Trial

## Abstract

**Background:**

The combination of nivolumab, a programmed death-1 (PD-1) targeted monoclonal antibody, with the cytotoxic T-lymphocyte antigen-4 (CTLA-4) targeted antibody, ipilimumab, represents a new standard of care in the first-line setting for patients with intermediate- and poor-risk metastatic renal cell carcinoma (mRCC) based on recent phase III data. Combining ipilimumab with nivolumab increases rates of grade 3 and 4 toxicity compared with nivolumab alone, and the optimal scheduling of these agents when used together remains unknown. The aim of the PRISM study is to assess whether less frequent dosing of ipilimumab (12-weekly versus 3-weekly), in combination with nivolumab, is associated with a favourable toxicity profile without adversely impacting efficacy.

**Methods:**

The PRISM trial is a UK-based, open label, multi-centre, phase II, randomised controlled trial. The trial population consists of patients with untreated locally advanced or metastatic clear cell RCC, and aims to recruit 189 participants. Participants will be randomised on a 2:1 basis in favour of a modified schedule of 4 doses of 12-weekly ipilimumab versus a standard schedule of 4 doses of 3-weekly ipilimumab, both in combination with standard nivolumab. The proportion of participants experiencing a grade 3 or 4 adverse reaction within 12 months forms the primary endpoint of the study, but with 12-month progression free survival a key secondary endpoint. The incidence of all adverse events, discontinuation rates, overall response rate, duration of response, overall survival rates and health related quality of life will also be analysed as secondary endpoints. In addition, the potential of circulating and tissue-based biomarkers as predictors of therapy response will be explored.

**Discussion:**

The combination of nivolumab with ipilimumab is active in patients with mRCC. Modifying the frequency of ipilimumab dosing may mitigate toxicity rates and positively impact quality of life without compromising efficacy, a hypothesis being explored in other tumour types such as non-small cell lung cancer. The best way to give this combination to patients with mRCC must be similarly established.

**Trial registration:**

PRISM is registered with ISRCTN (reference ISRCTN95351638, 19/12/2017).

**Trial status:**

At the time of submission, PRISM is open to recruitment and data collection is ongoing.

## Background

Kidney cancer is the 14th most common cancer worldwide with an estimated 400,000 new cases, and 175,000 attributable deaths, in 2018 [1]. The majority of kidney cancers (90%) are renal cell carcinomas (RCC), most of which (75%) are of the clear cell subtype [2].

For the past decade, the mainstay of treatment for patients with metastatic RCC (mRCC), has been in the form of small molecule, vascular endothelial growth factor receptor-targeted, tyrosine kinase inhibitors (VEGFR TKIs), such as sunitinib and pazopanib. Whilst most patients initially get a clinical benefit from VEGFR TKIs, acquired resistance is typically observed within months after starting therapy, with median overall survival (OS) in the region of 2 years [3, 4]. The introduction of checkpoint inhibitors, targeting the cytotoxic T-lymphocyte antigen 4 (CTLA-4) and programmed death-1 (PD-1) T-cell receptors, has transformed the treatment landscape for patients with mRCC. The anti-PD1 antibody nivolumab, for example, is a standard treatment option following failure of VEGFR TKI [5].

Recently (during the set-up of PRISM), initial results from the landmark phase III trial, CheckMate 214 (CM214) comparing nivolumab plus the anti-CTLA-4 antibody ipilimumab against standard of care sunitinib, have been reported. In total, 1096 participants with untreated metastatic clear cell RCC were randomised 1:1 to receive either sunitinib (50 mg 4 weeks on; 2 weeks off) or combination immunotherapy using a schedule of nivolumab 3 mg/kg (N3) plus ipilimumab 1 mg/kg (I1) every 3 weeks for 4 doses, followed by nivolumab 3 mg/kg every 2 weeks until disease progression or toxicity warranting treatment discontinuation [6]. Amongst the 77% of participants with intermediate- or poor-risk disease, as per International Metastatic Renal Cell Carcinoma Database Consortium (IMDC) criteria [7], median OS was not reached in the N3 + I1 arm and was 26.0 months in the sunitinib arm (HR 0.63, *p* < 0.001), establishing the combination as a new standard of care for the first-line treatment of patients with intermediate- and poor-risk mRCC [8]. Efficacy amongst favourable-risk participants formed an exploratory endpoint of the study and suggests improved response rates and PFS amongst sunitinib-treated patients, although these results should be interpreted with caution given the immature nature of the survival data and small sample size (*n* = 249). The reported toxicity associated with N3 + I1 was, however, significant. The rate of grade 3 or 4 treatment-related adverse events (AE) was 46% and led to treatment discontinuation in 22% of participants receiving the combination. In addition, high dose glucocorticoids (≥ 40 mg prednisone per day) were required in 35% of participants in order to manage immune-related AEs [6]. Until recently this combination treatment was not available in the UK, however it has gained approval for use in England through the national Cancer Drugs Fund in early April 2019 [9].

### Study rationale

Whilst CM214 has established the superior efficacy of combination nivolumab with ipilimumab over sunitinib in patients with intermediate- and poor-risk mRCC, optimal scheduling of these drugs has not been explored in this disease area. Additionally, improving our current understanding of why some patients respond to immunotherapy whilst many others derive no benefit is recognised as a research priority and translational studies focusing on predictive biomarkers forms a further important exploratory objective of the trial.

PRISM is a Phase II, open label, multi-centre, parallel group, randomised controlled trial. Our trial was designed before CM214 results were available and seeks to establish whether less frequent scheduling of ipilimumab is associated with improved tolerability in patients with mRCC in comparison to the schedule used in CM214, without adversely impacting activity (in comparison with historic control data).

Studies in other cancer types demonstrate ipilimumab dose and / or frequency can affect the toxicity and efficacy of treatment. In advanced melanoma, for example, 10 mg/kg ipilimumab every 3 weeks for 4 doses was associated with longer median OS but higher rates of grade 3 or 4 toxicity, in comparison to 3 mg/kg dosing [10]. A recently reported melanoma trial comparing I3 + N1 versus I1 + N3 again showed a favourable toxicity profile associated with a lower dose of ipilimumab, with no apparent difference in efficacy at a minimum follow-up of 12 months [11]. Varying doses and schedules of ipilimumab plus nivolumab have also been examined amongst 8 cohorts within the phase Ib CM012 study in patients with non-small cell lung cancer, including nivolumab 3 mg/kg q2W plus ipilimumab 1 mg/kg given either 6- (*n* = 39) or 12- (*n* = 38) weekly [12]. The schedule N3 (2-weekly) + I1 (6-weekly) was selected for phase III evaluation based on its safety and efficacy profile [13]. Thus, formal investigation of the scheduling of ipilimumab when given in combination with nivolumab in patients with mRCC is warranted.

It was not feasible to design the study for an internal comparison of efficacy given the phase II nature of the trial and the required sample size for such a comparison. As the trial was designed prior to CM214 results being available, PRISM is powered to compare activity of the modified schedule with historic sunitinib control data, however ancillary analyses will explore results in relation to CM214.

## Methods

### Trial objectives

#### Primary objective

The primary aim of the PRISM trial is to assess whether the proposed alternative scheduling of ipilimumab (12-weekly), when given in combination with nivolumab, warrants further consideration based on safety and efficacy, as defined by the proportion of participants experiencing a grade 3 or 4 adverse reaction (AR) within 12 months and 12-month progression-free survival (PFS).

#### Secondary objectives

Secondary objectives include assessment in each treatment arm of: incidence of adverse reactions; treatment discontinuation rates; overall response rate; duration of response; response rate post-progression (for those receiving treatment beyond progression); overall survival rates; and health-related quality of life (HR-QoL).

#### Exploratory objectives

To explore circulating and tissue-based predictive biomarkers of response to immunotherapy.

### Trial design

The study protocol and this manuscript have been written in accordance with standard protocol items: recommendations for interventional trials (SPIRIT) guidelines [14]. We have included a SPIRIT checklist as supplementary material (Additional file 1: Table S1).

**Fig. 1:**
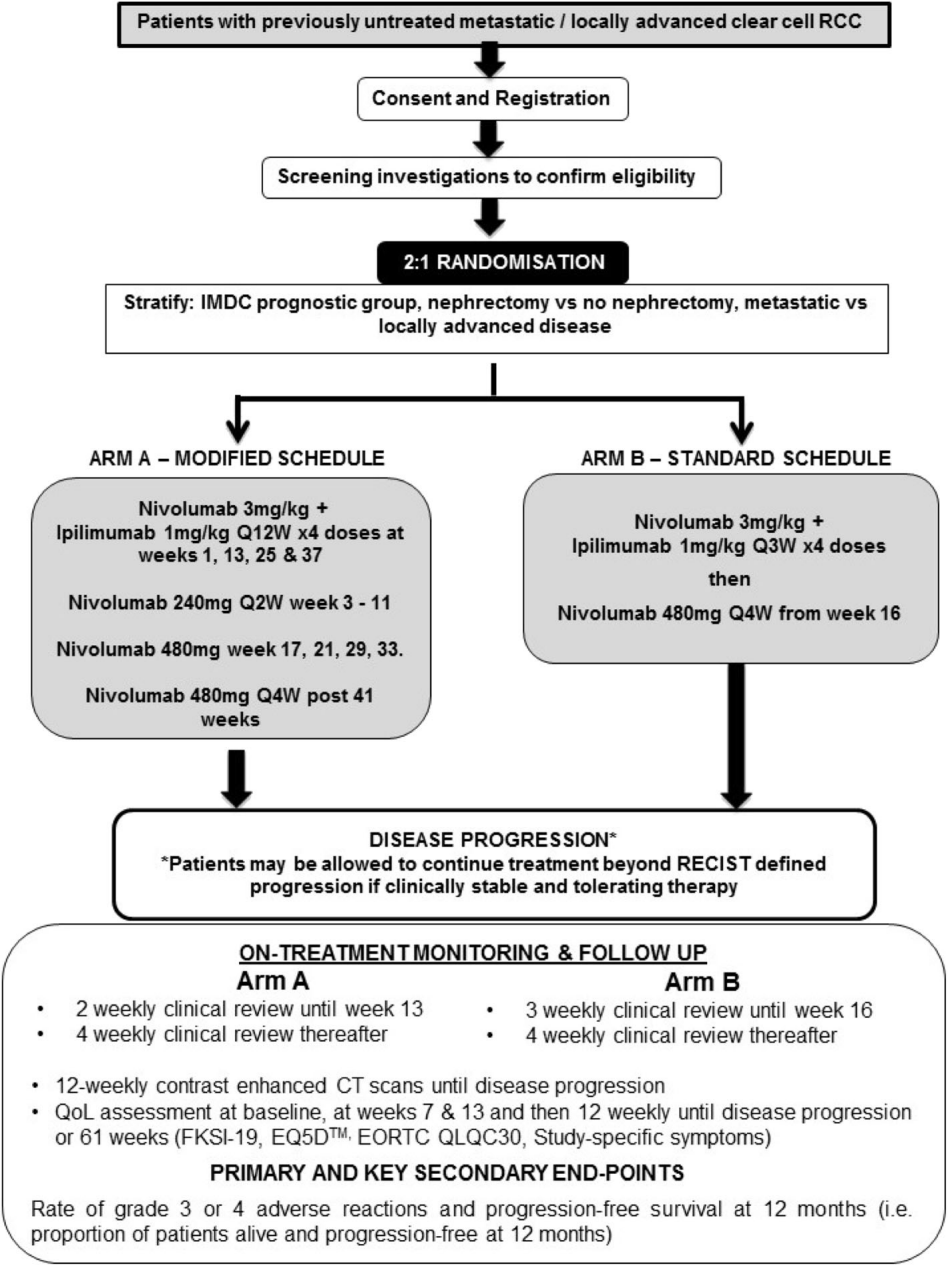
PRISM trial schema

**Fig. 2:**
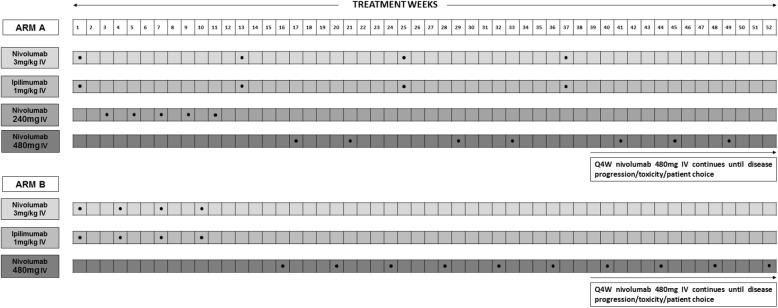
Treatment schedule. Frequency and dosing of nivolumab and ipilimumab for both arms of the study are shown

The PRISM trial is an open label, multi-centre, phase II, randomised controlled trial to explore the efficacy and safety of alternative reduced intensity scheduling of ipilimumab, when given in combination with nivolumab as first-line therapy, in patients with locally advanced or metastatic RCC. The trial will recruit 189 participants with individual randomisation on a 2:1 basis in favour of the modified schedule (see Fig. 1). The two arms of the trial are as follows (see Fig. 2):

- Arm A (modified schedule): 3 mg/kg nivolumab plus 1 mg/kg of ipilimumab every 12 weeks for 4 doses with:
  - 2-weekly 240 mg flat dose single agent nivolumab between the first and second combined doses, and
  - 4-weekly single agent nivolumab between the second and third, and third and fourth combined doses
  - Followed by 4-weekly 480 mg flat dose single agent nivolumab following the fourth combination dose, until disease progression/unacceptable toxicity/participant choice.
- Arm B (standard schedule): 3 mg/kg nivolumab intravenously plus 1 mg/kg of ipilimumab every 3 weeks for 4 doses. Followed by 4-weekly 480 mg flat dose single agent nivolumab following the fourth combination dose, until disease progression/unacceptable toxicity/participant choice.

Participants may receive treatment beyond first Response Evaluation Criteria In Solid Tumors (RECIST) v1.1 [15] defined progression based on investigator-assessed clinical benefit, tolerance of study drugs and stable performance status, as a minority of patients treated with immunotherapy may derive clinical benefit despite initial evidence of progressive disease. Trial treatment will be discontinued permanently upon documentation of further progression defined as the presence of any new lesion or an additional 10% increase in existing tumour burden from time of initial progression. Treatment will also be discontinued if treatment is delayed or interrupted for more than 6 weeks.

#### Quality of life evaluation

It is thought that nivolumab plus 12-weekly ipilimumab could be associated with favourable HR-QoL in comparison to standard 3-weekly dosing of ipilimumab. In order to evaluate treatment tolerability and its impact from a patient perspective, Patient-Reported Outcome Measures (PROM) to evaluate both overall HR-QoL and patient-reported AEs (physical symptoms of RCC and side effects of the treatments) will be collected. The severity and trajectory of AEs (symptoms or side-effects) of both treatment schedules will be reported, allowing comparison between clinician reported AEs (CTCAE V5.0) and patient-reported AEs using PROMs [16]. The primary outcome measure will be the summary score for HR-QoL of European Organisation for Research and Treatment of Cancer (EORTC) QLQ-C30. Secondary descriptive outcomes will include EQ-5D-5L™, physical symptoms of RCC (measured by Functional Assessment of Cancer Therapy Kidney Symptom Index (FKSI-19)) and known side-effects of ipilimumab and nivolumab measured by selected items from EORTC Quality of Life Group item bank [17]. We propose to use the item bank approach to cover expected toxicity of the investigational treatments, as due to the rapid introduction of new targeted cancer treatments there are no existing validated instruments that will cover the full range of AEs and using well developed and validated items from the item bank is a viable rapid alternative. PRO measures will be collected at baseline, weeks 7 and 13, then 12-weekly. The data collection will stop at disease progression or 61 weeks, whichever is earlier.

#### Translational study

The PRISM trial incorporates a strong translational element that seeks to explore circulating and tissue based predictive biomarkers of response to immunotherapy in patients with mRCC. The identification of such markers represents a research priority. Samples of plasma, serum and cell-free DNA will be collected in all consenting patients at baseline, weeks 7, 13 (selected to coincide with clinic visits) and at disease progression. Samples will be processed according to strict standard operating procedures (SOPs) defined within an associated trial-specific translational manual and all storage kit materials supplied centrally including barcoded blood tubes. In select centres, peripheral blood mononuclear cells (PBMCs) will also be collected from participants at the same time-points. Where available, an archival formalin-fixed paraffin embedded tissue block from nephrectomised participants will also be requested.

### Sample size

The trial will provide a comparison of tolerability between the modified schedule (Arm A) and standard schedule (Arm B) and will provide supportive data regarding the efficacy of the modified schedule (Arm A) in relation to historical control data with sunitinib [5]. To warrant further investigation, the modified schedule must meet both elements of the primary objective and show potential in terms of both tolerability and efficacy. In total, 189 patients (allowing for 5% attrition) will be recruited in order to adequately power both the toxicity and efficacy aspects of the study.

#### Toxicity

We expect approximately 40% of participants to experience a grade 3 or 4 AR within the initial 12 months of treatment when treated with the standard schedule (Arm B) [18]. To detect a clinically relevant reduction to 22% with the modified schedule (Arm A) (equivalent to a 45% relative reduction; odds ratio (OR) = 0.423), with 80% power at the two-sided 10% significance level, would require 178 participants, allowing for 5% attrition.

#### Efficacy

Assuming exponential survival, a median progression-free survival (PFS) with sunitinib of 9 months [5], (equivalent to 39.7% patients progression-free at 12 months) and targeting a minimum clinically relevant hazard ratio of 0.73 (corresponding to a median PFS in the modified Arm A schedule of 12.3 months, or 50.9% progression-free at 12 months) 120 participants would be required in the modified schedule arm to give 80% power at the one-sided 5% significance level. With 2:1 randomisation, a total of 189 patients will need to be recruited to allow for 5% attrition. Comparison with historical control data is based on data available at the time of design from the COMPARZ study [5], however additional unpowered analyses will be considered in light of the CM214 results now being available.

### Consent, eligibility, screening and registration

Potential participants from participating NHS hospitals in the UK will be provided with verbal and written information about the trial and given as long as required to consider participation. Assenting patients will provide written informed consent and be registered to the trial via a central automated 24-h system (provided by University of Leeds) prior to any trial specific assessments being conducted. Participants can also optionally consent to take part in HR-QoL and biomarker sub-studies. Participants retain the right to withdraw at any time without giving reasons and without their further treatment being prejudiced.

A full list of inclusion and exclusion criteria can be found in Table 1. In short, patients aged 18 or over with advanced or metastatic clear cell RCC, RECIST measurable disease and a Karnofsky Performance Status of at least 70% will be eligible. All IMDC risk-groups, including those with favourable-risk disease, are eligible. Patients will not be eligible if they have undergone prior systemic anti-cancer treatment or if they have active, known or suspected autoimmune disease. Eligibility waivers will not be granted in this trial.

**Table 1:** Eligibility Criteria

Inclusion criteria	
Aged 18 years or over	
Diagnosed with advanced (not amenable to curative surgery) or metastatic RCC	
Histopathologically confirmed clear cell renal cell cancer (or with a component of clear cell)	
Evidence of measurable disease as per RECIST v1.1	
Life expectancy of ≥6 months	
Karnofsky Performance Status (KPS) of at least 70%	
Required laboratory values within 14 days prior to randomisation: • WBC ≥ 2 × 10^9^/L • Neutrophils ≥1.5 × 10^9^/L • Platelets ≥100 × 10^9^/L • Haemoglobin > 9.0 g/dL • Serum creatinine ≤1.5 x ULN or calculated creatinine clearance (CrCl) ≥ 40 mL/min (Cockcroft and Gault or Wright formula may be used according to local practice) • AST and ALT ≤3 x ULN • Total Bilirubin ≤1.5 x ULN (except subjects with Gilbert Syndrome, who can have total bilirubin < 50 μmol/L)	
Able to give written informed consent and willing to follow trial protocol	
Women of childbearing potential (WOCBP) must: • Use appropriate method(s) of contraception for 23 weeks after the last dose of investigational drug. • Have a negative serum or urine pregnancy test (minimum sensitivity 25 IU/L or equivalent units of HCG) before randomisation.	
Men who are sexually active with WOCBP must agree to use any contraceptive method with a failure rate of less than 1% per year. Men who are sexually active with WOCBP must agree to adhere to contraception for a period of 31 weeks after the last dose of investigational product.	
Exclusion criteria	
Pregnant or breast feeding females.	
Prior systemic therapy for RCC (previous participation in adjuvant studies allowed, providing the patient was on the observation/placebo arm – this may require unblinding of the patient)	
Participants who have undergone any prior systemic anti-cancer treatment, including with an anti-PD-1, anti-PD-L1, anti-PD-L2, anti-CTLA-4 antibody, or any other antibody or drug specifically targeting T-cell co-stimulation or immune checkpoint pathways (previous participation in adjuvant studies allowed, providing the patient was on the observation/placebo arm – this may require unblinding of the patient)	
Prior malignancy active within the previous 3 years except for locally curable cancers that have been apparently cured, such as basal or squamous cell skin cancer, superficial bladder cancer, or carcinoma in situ of the prostate, cervix, or breast.	
Participants who test positive for hepatitis B virus surface antigen (HBV sAg) or hepatitis C virus ribonucleic acid (HCV antibody) indicating acute or chronic infection.	
Participants who test positive for human immunodeficiency virus (HIV) or have known acquired immunodeficiency syndrome (AIDS).	
Untreated brain metastases or brain metastases treated only with whole brain radiotherapy. (Patients are eligible if previous brain metastases treated with complete surgical resection, Stereotactic Brain Radiation Therapy (SBRT), or gamma knife with no subsequent evidence of progression and asymptomatic).	
Active, known or suspected autoimmune disease. (Subjects are permitted to enrol if they have vitiligo, type I diabetes mellitus, residual hypothyroidism due to autoimmune condition only requiring hormone replacement, psoriasis not requiring systemic treatment, or conditions not expected to recur in the absence of an external trigger).	
Patients should be excluded if they have a condition requiring systemic treatment with either corticosteroids (> 10 mg daily prednisone or equivalent) or other immunosuppressive medications within 14 days of study drug administration. (Inhaled or topical steroids and adrenal replacement doses > 10 mg daily prednisone equivalents are permitted in the absence of active autoimmune disease).	
Uncontrolled adrenal insufficiency.	
Any serious or uncontrolled medical disorder that, in the opinion of the investigator, may increase the risk associated with study participation or study drug administration, impair the ability of the subject to receive protocol therapy, or interfere with the interpretation of study results.	
Palliative radiotherapy less than 14 days prior to first dose of study drug.	
Any history of hypersensitivity to any of the trial medications or excipients.	
Poorly controlled or serious medical or psychiatric illness that, in the Investigator’s opinion, is likely to interfere with participation and/or compliance in this clinical trial.	

Screening assessments for eligibility will include: Computed tomography (CT) chest, abdomen and pelvis; electrocardiogram (ECG); physical exam and medical history; full blood count (FBC); urea and electrolytes (U&Es); liver function tests (LFTs); hepatitis B/C and human immunodeficiency virus (HIV) tests; calculated creatinine clearance (CrCI); Karnofsky performance status (KPS); IMDC prognostic group classification; where applicable, pregnancy test. Consenting patients will also be asked to complete baseline HR-QoL questionnaires. Those assenting will also give samples of serum and plasma (and at selected sites only, peripheral blood mononuclear cells [PBMCs]).

### Randomisation

Randomisation will be performed following confirmation of eligibility (and prior to any trial treatment) via a central automated 24-h system (provided by University of Leeds) to either the modified schedule or the standard schedule on a 2:1 allocation ratio in favour of the modified schedule. A computer–generated minimisation programme incorporating a random element generated by the independent CTRU Statisticians will be used to ensure treatment groups are well balanced proportionately with respect to: IMDC prognostic group (favourable/intermediate/poor risk); nephrectomy status (nephrectomy/no nephrectomy); and disease type (metastatic/locally advanced). Irrespective of their randomised allocation, participants should commence therapy within 14 days of randomisation.

### Assessments

A schedule of assessments is provided as supplementary material in (Additional file 2: Table S2).

#### During treatment

Due to the difference in treatment schedules, participants in Arm A (modified schedule) will be assessed every 2 weeks for the first 12 weeks while those in Arm B (standard schedules) will be assessed every 3 weeks until week 16. Thereafter, all participants will be assessed 4-weekly. Each assessment will include: clinical assessment; adverse event reporting; LFTs; FBC; U&Es; thyroid function test (TFT); glucose; calcium; amylase; and cortisol. PROMs will be completed at baseline and weeks 7, 13, and then 12-weekly until 61 weeks or until disease progression, whichever is earlier. Assenting participants will give samples of serum and plasma (and at selected sites only, PMBCs) at baseline, weeks 7, 13 and at disease progression.

The differential reporting between trial arms is not felt to be a large concern due to: i) the short time lapse between assessments; ii) there being only 1 week’s difference in the reporting period between trial arms; iii) follow-up being 4-weekly for both trial arms for the majority of the trial; and iv) the primary and key secondary endpoints being over 12 months. In addition, this differential follow-up is in favour of the standard schedule (Arm B) due to more frequent reporting in Arm A (modified schedule), and is therefore conservative.

All participants will receive radiological assessment according to RECIST criteria using contrast enhanced CT of the chest, abdomen and pelvis every 12 weeks until disease progression or treatment discontinuation (whichever occurs later).

#### Follow up

The duration of follow-up for individual participants will vary. Every participant will be followed up with radiological assessments until disease progression or treatment discontinuation, whichever occurs later. Safety follow-up will continue for 100 days after the last dose of study drug or death, whichever occurs earlier. A follow-up visit will be conducted at 30 days post last dose of trial treatment where assessments will include: clinical assessment; adverse event reporting; FBC; U&Es; LFTs; TFTs; and cortisol. Participants will be followed up for overall survival 12 months post last patient randomised via centre follow-up.

### Outcomes

#### Primary endpoint

The primary endpoint is the proportion of participants experiencing a grade 3 or 4 adverse reaction within the initial 12 months of treatment as graded by CTCAE v5.0.

#### Key secondary endpoint

Efficacy forms a key secondary endpoint of the study and will be based on the proportion of participants alive and progression-free at 12 months. Progression-free survival will be calculated from the date of randomisation to first documented evidence of disease progression (based on RECIST v1.1 criteria) or death. This landmark survival endpoint provides a pragmatic and timely readout of efficacy in this phase II trial and was selected before the results of the CM214 trial were available. Since the CM214 trial has now reported, additional exploratory analyses will be considered (such as the presentation of summary statistics for PFS at 12 m by prognostic group) and will be pre-specified prior to the final analysis being conducted.

#### Secondary endpoints

- *Safety and toxicity*: adverse reactions (ARs), serious adverse events (SAEs), serious adverse reactions (SARs) and suspected unexpected serious adverse reactions (SUSARs) as measured throughout the trial and graded by common terminology criteria for adverse events (CTCAE) v5.0;
- *Treatment tolerability*: the proportion of participants who experience a treatment delay, the average number of treatment delays per participant and the duration of delays as measured over the course of trial treatment;
- *Treatment discontinuation*: rates of treatment discontinuation at any point during trial treatment and reasons;
- *Overall response*: the proportion of participants with partial response or complete response (PR or CR) as their best response to treatment prior to first-progression as defined by RECIST v1.1;
- *Duration of response*: calculated from first observation of at least PR until disease progression (as defined by RECIST v1.1) or death;
- *Overall survival:* calculated from the date of randomisation to the date of death from any cause;
- *Response rate for treatment beyond progression:* the proportion of participants showing a partial or complete best response (PR or CR) as defined by RECIST v1.1 when treated beyond progression.
- *Health-related Quality of Life:* assessed using the FKSI-19; the EORTC QLQ-C30; study-specific symptoms and the EQ-5D-5L^TM^ and scored using the corresponding scoring manuals.

### Statistical methods and analysis

Analyses will be conducted following modified intention-to-treat principles (unless otherwise stated a priori) meaning participants will be analysed in the group to which they were randomised regardless of compliance or cross-over. Participants will be included in the primary and key secondary analyses provided they have received at least one dose of trial treatment and have provided the relevant outcome data.

The primary analysis will examine differences in the proportion of participants experiencing a grade 3 or 4 AR within the initial 12 months of treatment between trial arms using a logistic regression model, adjusting for minimisation factors (IMDC prognostic group, nephrectomy status, disease type); odds ratios will be presented alongside corresponding confidence intervals (CI). Pre-specified sensitivity analyses may be conducted as appropriate.

Should the primary analysis show a reduction in toxicity for the modified schedule (Arm A) compared with the standard schedule (Arm B), the formal key secondary analysis will be conducted. If the lower limit of the 90% CI for the proportion of participants alive and progression-free at 12 months in the modified schedule only (Arm A) excludes the rate of no interest based on historical (sunitinib-treated) control data (39.7%), the modified schedule will be deemed to have sufficient activity in line with that expected in the CM214 trial. The trial will provide supportive information rather than definitive conclusions of superiority of the modified arm to sunitinib.

Secondary endpoints will be analysed using summary statistics alongside confidence intervals where appropriate. All analyses will be fully detailed in a statistical analysis plan prior to being undertaken.

### Trial conduct and oversight

Data will be collected via electronic case report forms (eCRF). The trial will be conducted in accordance with the principles of Good Clinical Practice (GCP) and in line with the relevant Research Governance Framework within the UK through adherence with CTRU standard operating procedures (SOPs). An independent Data Monitoring and Ethics Committee (DMEC) will be established to review safety data on a regular basis to identify any safety concerns or trends. An independent Trial Steering Committee (TSC) will periodically review safety data and discuss recommendations made by the DMEC.

## Discussion

Exploration of drug dosing schedules is an important, yet perhaps over-looked, factor in optimising treatment for patients with cancer. Alternative scheduling of drugs may lead to improvement in efficacy and/or tolerability, as well as in terms of cost-effectiveness. Studies demonstrating improved tolerability for sunitinib taken on a 2 weeks on, 1 week off basis compared to the standard 4:2 schedule provide a good example of this [19]. Similarly, enthusiasm for the rapid adoption of immune-oncology agents into clinical practice must, in parallel, be matched by careful and robust examination of dosing and schedule, as well as duration of therapy, if efficacy, tolerability, QoL and cost-effectiveness are to be maximised. The phase III CM214 study has formally established the efficacy of the combination of nivolumab plus ipilimumab (using standard 3-weekly ipilimumab) in patients with mRCC. As oncologists begin to adopt the regimen into routine clinical practice, the PRISM trial has been designed with the important aim of establishing whether reduced intensity scheduling of ipilimumab is associated with reduced toxicity and improved QoL for patients, without compromising activity. The study has been designed pragmatically, since formal demonstration of non-inferiority of the experimental schedule to the current standard in terms of efficacy would not have been feasible given the required sample size. The results from our trial may lead to change in clinical practice in patients with mRCC and have implications for the scheduling of ipilimumab in other tumour settings when used in combination with nivolumab. Additionally, as treatment options continue to burgeon in mRCC, PRISM meets a research priority by aiming to identify predictive biomarkers of response to immunotherapy in this patient population, helping lead the way towards personalised medicine in this area.

## Supplementary information

**Additional file 1: Table S1.** SPIRIT Checklist.

**Additional file 2:** **Table S2.** Schedule of Assessment.

## Data Availability

Not applicable.

## Abbreviations

AE: Adverse event
ALT: Alanine transaminase
AR: Adverse reaction
AST: Aspartate transaminase
CI: Confidence interval
CM214: Checkmate 214 trial
CR: Complete Response
CrCI: Creatinine clearance
CT: Computed tomography
CTCAE: Common Terminology Criteria for Adverse Events
CTLA-4: Cytotoxic T-lymphocyte antigen 4
CTRU: Clinical Trials Research Unit
DMEC: Data monitoring and ethics committee
ECG: Electrocardiogram
eCRF: Electronic case report form
EORTC: European Organisation for Research and Treatment of Cancer
EQ-5D: EuroQoL 5-dimension
EU: European Union
FBC: Full Blood Count
FDA: Food and Drug Administration
FKSI-19: Functional assessment of cancer therapy kidney symptoms index-19
GCP: Good Clinical Practice
HIV: Human immunodeficiency virus
HR: Heart rate
HR-QoL: Health related quality of life
IMDC: International Metastatic RCC Database Consortium
KPS: Karnofsky performance status
LFT: Liver Function Tests
mITT: Modified intention to treat
mRCC: Metastatic renal cell carcinoma
OR: Odds ratio
ORR: Overall response rate
OS: Overall survival
PBMCs: Peripheral blood mononuclear cells
PD: Progressive disease
PD-1: Programmed death-1
PFS: Progression-free survival
PR: Partial response
PRO: Patient-Reported Outcome
QoL: Quality of Life
RCC: Renal cell carcinoma
REC: Research Ethics Committee
RECIST: Response Evaluation Criteria In Solid Tumors
SAE: Serious adverse event
SAR: Serious adverse reaction
SD: Stable disease
SOPs: Standard operating procedures
SUSAR: Suspected unexpected serious adverse reaction
TFT: Thyroid Function Tests
TKI: Tyrosine Kinase Inhibitor
TSC: Trial Steering Committee
U&Es: Urea and Electrolytes
UK: United Kingdom
VEGF: Vascular endothelial growth factor
WBC: White blood cell (count)
