# Supplementary material for: PRISM protocol: a randomised phase II trial of nivolumab in combination with alternatively scheduled ipilimumab in first-line treatment of patients with advanced or metastatic renal cell carcinoma
Source: BMC Cancer. 2019 Nov 14;19:1102. doi: 10.1186/s12885-019-6273-1 (PMC6854710; doi:10.1186/s12885-019-6273-1)
Supplement: Supplementary file 2 — Additional file 2: Table S2. Schedule of Assessment. [file 12885_2019_6273_MOESM2_ESM.docx]

|  |  | **Screening and consent** | **Baseline** | | **Allocation** | **Treatment** | | | | | | | | | | | | | | | | **End of Treatment** | **Unscheduled** |
| --- | --- | --- | --- | --- | --- | --- | --- | --- | --- | --- | --- | --- | --- | --- | --- | --- | --- | --- | --- | --- | --- | --- | --- |
|  |  | Prior to registration | After registration and within 28 days prior to randomisation^Ɨ^ | After registration and within 14 days prior to randomisation^Ɨ^ |  | **Week** | | | | | | | | | | | | | | | 4-weekly post combination phase* | 30 and 100 days post last dose of study drug |  |
|  |  |  |  |  |  | 1 | 3 | 4 | 5 | 7 | 9 | 10 | 13 | 16 | 17 | 21 | 25 | 29 | 33 | 37 |  |  |  |
|  | Informed Consent | X |  |  |  |  |  |  |  |  |  |  |  |  |  |  |  |  |  |  |  |  |  |
|  | Screening data | X |  |  |  |  |  |  |  |  |  |  |  |  |  |  |  |  |  |  |  |  |  |
|  | Eligibility |  | X |  |  |  |  |  |  |  |  |  |  |  |  |  |  |  |  |  |  |  |  |
|  | Baseline assessments^з^ |  |  | X |  |  |  |  |  |  |  |  |  |  |  |  |  |  |  |  |  |  |  |
|  | Pregnancy test^α^ |  | X |  |  | X |  |  |  |  |  |  |  |  |  |  |  |  |  |  |  |  | X^¶^ |
|  | KPS and IMDC classification |  |  | X |  |  |  |  |  |  |  |  |  |  |  |  |  |  |  |  |  |  |  |
|  | Translational blood samples^~^ |  |  | X |  |  |  |  |  | X |  |  | X |  |  |  |  |  |  |  |  |  | X** |
|  | Archival tissue block |  |  |  |  |  |  |  |  |  |  |  |  |  |  |  |  |  |  |  |  |  | X^Ω^ |
|  | CT chest, abdomen and pelvis |  | X |  |  | X |  |  |  |  |  |  | X |  |  |  | X |  |  | X |  |  | X^ϕ^ |
|  | ECG |  | X |  |  |  |  |  |  |  |  |  |  |  |  |  |  |  |  |  |  |  |  |
|  | Randomisation |  |  |  | X |  |  |  |  |  |  |  |  |  |  |  |  |  |  |  |  |  |  |
| **Arm A** | **Pre-treatment assessments** | |  |  |  |  |  |  |  |  |  |  |  |  |  |  |  |  |  |  |  |  |  |
|  | Clinical assessment/Vital signs |  |  |  |  | X | X |  | X | X | X |  | X |  | X | X | X | X | X | X | X | X |  |
|  | AEs |  |  |  |  | X | X |  | X | X | X |  | X |  | X | X | X | X | X | X | X | X |  |
|  | Dose delay details |  |  |  |  | X | X |  | X | X | X |  | X |  | X | X | X | X | X | X | X |  |  |
|  | LFTs |  |  |  |  | X | X |  | X | X | X |  | X |  | X | X | X | X | X | X | X | X |  |
|  | Other laboratory tests |  |  |  |  | X |  |  | X |  | X |  | X |  | X | X | X | X | X | X | X | X |  |
|  | **Treatment details** |  |  |  |  |  |  |  |  |  |  |  |  |  |  |  |  |  |  |  |  |  |  |
|  | Nivolumab 3mg/kg & Ipilimumab 1mg/kg |  |  |  |  | X |  |  |  |  |  |  | X |  |  |  | X |  |  | X |  |  |  |
|  | Nivolumab (240mg/480mg) |  |  |  |  |  | X^ᴫ^ |  | X^ᴫ^ | X^ᴫ^ | X^ᴫ^ |  |  |  | X^$^ | X^$^ |  | X^$^ | X^$^ |  | X^$^ |  |  |
| **Arm B** | **Pre-treatment assessments** | |  |  |  |  |  |  |  |  |  |  |  |  |  |  |  |  |  |  |  |  |  |
|  | Clinical assessment/Vital signs |  |  |  |  | X |  | X |  | X |  | X | X | X |  |  |  |  |  |  | X | X |  |
|  | AEs |  |  |  |  | X |  | X |  | X |  | X |  | X |  |  |  |  |  |  | X | X | X** |
|  | Dose delay details |  |  |  |  | X |  | X |  | X |  | X |  | X |  |  |  |  |  |  | X |  |  |
|  | LFTs |  |  |  |  | X |  | X |  | X |  | X | X | X |  |  |  |  |  |  | X | X |  |
|  | Other laboratory tests |  |  |  |  | X |  | X |  | X |  | X | X | X |  |  |  |  |  |  | X | X |  |
|  | **Treatment details** |  |  |  |  |  |  |  |  |  |  |  |  |  |  |  |  |  |  |  |  |  |  |
|  | Nivolumab 3mg/kg & Ipilimumab 1mg/kg |  |  |  |  | X |  | X |  | X |  | X |  |  |  |  |  |  |  |  |  |  |  |
|  | Nivolumab 480mg |  |  |  |  |  |  |  |  |  |  |  |  | X |  |  |  |  |  |  | X |  |  |
|  | QoL Outcomesǂ |  | X |  |  |  |  |  |  | X |  |  | X |  |  |  | X |  |  | X | X^^^ |  |  |
|  | SAEs |  |  |  |  |  |  |  |  |  |  |  |  |  |  |  |  |  |  |  |  |  | X |
|  | First disease progression |  |  |  |  |  |  |  |  |  |  |  |  |  |  |  |  |  |  |  |  |  | X |
|  | Second disease progression |  |  |  |  |  |  |  |  |  |  |  |  |  |  |  |  |  |  |  |  |  | X |
|  | Ɨ Some assessments may have been completed prior to registration as part of routine standard of care within the timelines specified, if this is the case, they do not need to be repeated specifically for the trial; * Arm A patients, 4-weekly assessments correspond to 4-weekly post week 37, and for Arm B, this corresponds to 4-weekly assessments post week 16; з Baseline assessments including: Physical examination/vital signs, medical history, laboratory tests; α for women of child bearing potential only; ¶ Pregnancy test also monthly throughout treatment and for 31 weeks following if woman of child bearing potential; ~ For those providing consent for translational elements of trial; ** At first, and (if applicable) second disease progression; Ω Archival tissue collected post randomisation if available and consenting; Φ 12 weekly CT scans until disease progression or death; ᴫ 240mg nivolumab; $ 480mg nivolumab; ǂ QoL outcomes as measured by EORTC QLQ-C30; FKSI-19; Study specific symptoms; EQ-5D-5L; ^ Post-combination phase weeks 49 and 61 only. | | | | | | | | | | | | | | | | | | | | | | |

## Additional file 2: Table S2: Schedule of Assessment
